# 

**Published:** 1848-08

**Authors:** 


					﻿Notice.—We beg to call the attention of our readers to the fact that this
number of the journal contains eight additional pages. They have been
added in consequence of the length of the Report of the Trial for Mal-prac-
tice, in order that the publication of that report may not interfere with the
usual variety of matter.
A word to Patrons.—Our patrons, we trust, will observe that our
Journal has recently entered upon a new year—the fourth year of its
existence. The effort to render its monthly visitations acceptable, costs us
a degree of labor which they know little of, who have never been engaged
in a similar undertaking. But this labor we shall continue to bestow cheer-
ily, provided we have good reasons to believe that the work is fulfilling,
m sorrfe measure, the useful objects for which it was established- Fbr the
flattering expressions with which we have been honored, especially from
our brethren of the medical corps editoriale, we are deeply grateful, at the
same time, being duly sensible, hpw much' of these expressions is to be
attributed to their kindness, rather than to our deserts. To those subscri-.
bers who have testified both by words, and in a more substantial mode,
their sense of the value of the work, we tender our grateful acknowledge-
ments. We have not space to add more, nor are we disposed to occupy
much of our pages with these matters. We will close by the word.to
which the foregoing is prefatory:—The present is the most favorable period
of the year for the friends of the Journal to renew subscriptions, pay up
arrears, and use exertions to extend its circulation.
Books.—Bibliographical favors already received, have not all, as yet,
had due attention. Several books remain, which will be reviewed' or
noticed in our next number.

				

